# Streptomyces-Fungus Co-Culture Enhances the Production of Borrelidin and Analogs: A Genomic and Metabolomic Approach

**DOI:** 10.3390/md22070302

**Published:** 2024-06-28

**Authors:** Tan Liu, Xi Gui, Gang Zhang, Lianzhong Luo, Jing Zhao

**Affiliations:** 1 College of Ocean and Earth Science, Xiamen University, Xiamen 361005, China; liutan@stu.xmu.edu.cn (T.L.); xigui@stu.xmu.edu.cn (X.G.); 2 Xiamen Key Laboratory of Marine Medicinal Natural Product Resources, Xiamen Medical College, Xiamen 361005, China; zg@xmmc.edu.cn (G.Z.); llz@xmmc.edu.cn (L.L.)

**Keywords:** co-culture, molecular network, gene cluster, secondary metabolites, bioactivity

## Abstract

The marine Streptomyces harbor numerous biosynthetic gene clusters (BGCs) with exploitable potential. However, many secondary metabolites cannot be produced under laboratory conditions. Co-culture strategies of marine microorganisms have yielded novel natural products with diverse biological activities. In this study, we explored the metabolic profiles of co-cultures involving *Streptomyces* sp. 2-85 and *Cladosporium* sp. 3-22—derived from marine sponges. Combining Global Natural Products Social (GNPS) Molecular Networking analysis with natural product database mining, 35 potential antimicrobial metabolites annotated were detected, 19 of which were exclusive to the co-culture, with a significant increase in production. Notably, the Streptomyces-Fungus interaction led to the increased production of borrelidin and the discovery of several analogs via molecular networking. In this study, borrelidin was first applied to combat *Saprolegnia parasitica*, which caused saprolegniosis in aquaculture. We noted its superior inhibitory effects on mycelial growth with an EC_50_ of 0.004 mg/mL and on spore germination with an EC_50_ of 0.005 mg/mL compared to the commercial fungicide, preliminarily identifying threonyl-tRNA synthetase as its target. Further analysis of the associated gene clusters revealed an incomplete synthesis pathway with missing malonyl-CoA units for condensation within this strain, hinting at the presence of potential compensatory pathways. In conclusion, our findings shed light on the metabolic changes of marine Streptomyces and fungi in co-culture, propose the potential of borrelidin in the control of aquatic diseases, and present new prospects for antifungal applications.

## 1. Introduction

Sponges serve as reliable sources for isolating novel marine actinomycete species and natural products. Chemical defense is an important adaptation for sponges to deter predators and competitors, with diverse bioactive compounds identified such as sterols, nucleosides, cyclic peptides, and alkaloids [1]. Interestingly, these compounds are not exclusively synthesized by sponges but by their microbial symbionts [2]. Marine actinomycetes are recognized as a rich source for marine drug discovery. Adapted to the unique conditions of marine environments, such as high pressure, high salinity, and nutrient deficiencies, marine actinomycetes produce secondary metabolites distinct from their terrestrial counterparts [3]. *Streptomyces* spp. produce over 30 secondary metabolites on average, making them prolific secondary metabolite producers, which contributes to over 80% of bioactive compound production from natural sources [4]. However, microbial genome sequence analyses indicate that a single genome may contain 20–80 distinct BGCs, most of which are cryptic and cannot be expressed under standard laboratory culture conditions [5]. This suggested that the metabolic capabilities of bacteria exceeded what could be demonstrated in the laboratory due to severe gene silencing or low synthesis yields, making it challenging for analytical methods to detect these substances.

The One Strain Many Compounds (OSMAC) strategy has been proven to be a promising tool for activating silent BGCs by altering pH and temperature, controlling the supply of oxygen, carbon, nitrogen, or phosphorus sources, and adding metal ions or inducers [6]. For example, the OSMAC strategy was used to identify Terrosamycins A and B, as well as two cyclic polyethers with anti-breast cancer activity from *Streptomyces* sp. RKND004 [7]. In addition, co-culture is another promising approach to activating novel secondary metabolites in marine actinomycetes by mimicking microbial interactions in natural environments. This approach stimulates the expression of cryptic BGCs by promoting interactions, defense, communication, and metabolite competition [8]. Moreover, marine sponges, associated with rich microorganisms, serve as natural microbial fermenters, providing abundant microbial resources and environments for co-culture. Common modes of co-culture include bacteria-bacteria, fungus-fungus, and bacterial-fungal co-cultures. For example, co-culturing *Janthinobacterium* sp. ZZ145 and ZZ148 yielded janthinopolyenemycins A and B, active against *Candida albicans* [9]; co-culturing *Talaromyces aculeatus* (Genbank no. KY76541) and *Penicillium variabile* HXQ-H-1 produced four novel polyketides toxic to cancer cell lines [10]; and co-culturing *Streptomyces lividans* TK24 with *Fusarium tricinctum* yielded four new naphthoquinone dimers, fusatricinones A–D [11].

The integration of genomic mining with metabolomic analysis has significantly accelerated natural product discovery. Genome mining has emerged as a powerful tool for estimating the genetic potential of strains, involving the scanning of the genome to identify BGCs responsible for secondary metabolites. Various tools, such as antiSMASH, are available for accurate prediction and analysis of BGCs and pathways [12]. Additionally, Mass spectrometry (MS)-based metabolomics plays a crucial role in studying natural products [13]. FBMN (Feature-Based Molecular Networking), a key analytical tool on the GNPS (Global Natural Products Social) Molecular Networking platform, organizes molecular ions into networks, streaming workflows and facilitating molecule annotation and analogs discovery [14]. By integrating MS/MS databases and molecular networks, correlating genomic and metabolite data deepens the understanding of BGCs, enhances structural elucidation accuracy, and reveals biosynthetic pathways and potential bioactive compounds. For instance, combinatorial genomics-metabolomics analysis of the marine strain led to the identification of novel bioactive compounds, including spirindamycins E and F, as well as two new α-pyrones, lagunapyron [15].

*Saprolegnia parasitica* is an aquatic pathogen posing a tremendous threat to the global aquaculture industry that manifests itself as grayish-white filamentous mycelial spots on the body or fins of fish [16]. The traditional treatment is to control it with malachite green, which was banned in 2002 due to its high carcinogenicity and toxicity [17]. Therefore, there is an urgent need to find more alternative treatments. In this study, we assessed the BGCs diversity of the Streptomyces strain and analyzed the secondary metabolite profiles of marine Streptomyces and fungal co-cultures. By integrating LC-MS/MS spectra, bioactivity assays, GNPS (Global Natural Products Social) molecular networks, exploration of natural product databases, isolation and purification of active compounds, and preliminary exploration of inhibitory mechanisms, we provided a comprehensive analysis of co-cultural biosynthetic diversity, demonstrating a promising potential for discovering bioactive compounds to combat aquatic pathogens.

## 2. Results

### 2.1. BGCs Prediction of Streptomyces sp. 2-85

*Streptomyces* sp. 2-85 demonstrated the closest phylogenetic relationship with *Streptomyces rochei* S32, sharing 99.8% homology based on the 16 S rRNA gene sequence (Figure S1). The genome of *Streptomyces rochei* S32 was analyzed using antiSMASH 7.0 to assess its ability to form biosynthetic gene clusters (BGCs). A total of 31 BGCs related to secondary metabolites were identified (Table S1), mainly including type I–III polyketide synthase (PKS I–III, 6), nonribosomal peptide synthetases (NRPS, 1), hybrid (6), terpene (7), lanthipeptide (6), melanin (1), indole (2), ectoine (1), and saccharide (1).

The predicted BGCs account for 16.39% (1318 Mb) of the entire bacterial chromosomal genome, of which 13 belong to PKS, NRPS, or mixed PKS-NRPS types; 28 BGCs show varying degrees of similarity to known BGCs; 11 BGCs show no more than 60% similarity to known BGCs; and the remaining 3 BGCs could not be recognized, suggesting that the strain still has the potential to produce novel natural products.

### 2.2. Anti-Microbial and Antifungal Activity Assessment and Variations in Secondary Metabolite Profiles under Different Cultivation Conditions

The extract from *Streptomyces* sp. 2-85 mono-culture did not exhibit the expected levels of secondary metabolites, which was inconsistent with its genomic biosynthetic potential. Therefore, we employed metabolomic approaches to co-culture actinomycetes and fungi under different media and pH conditions and observed changes in the metabolites of co-cultures, aiming to elucidate the mechanism underlying the enhanced antibacterial effect of co-culture.

**Figure 1:**
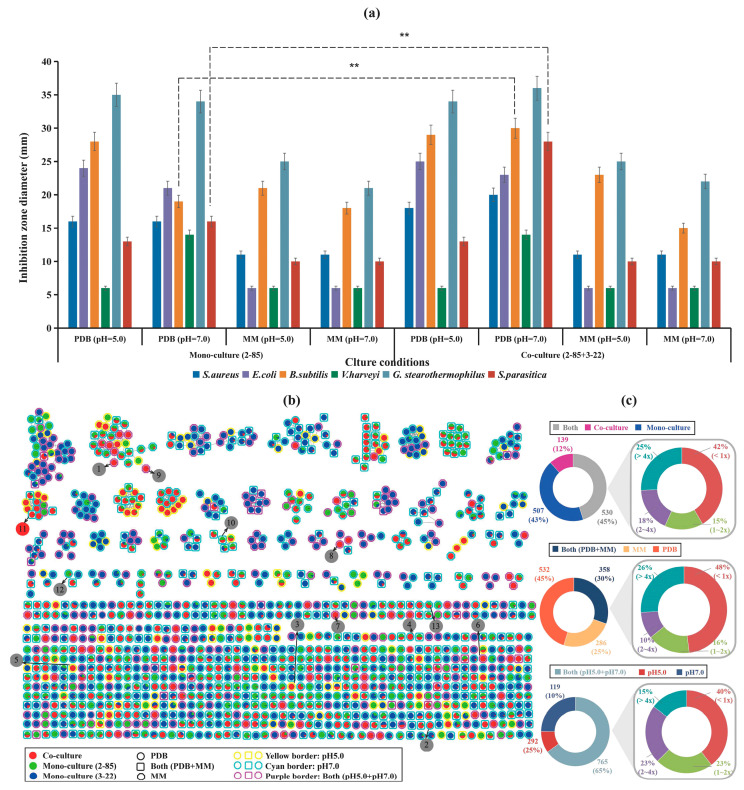
(**a**) Anti-microbial bioassay and comparative analysis under different culture conditions (** *p* ≤ 0.01); (**b**) Global natural products social (GNPS) molecular networking analysis (-ion mode) of LC-MS/MS of 12 fermentation extracts from mono- *Streptomyces* sp. 2-85 and *Cladosporium* sp. 3-22 and their co-cultures in 2 media and different pH; (**c**) Fold change distribution of the metabolites signal intensity among different cultures.

The antibacterial activity of *Streptomyces* sp. 2-85 and *Cladosporium* sp. 3-22 was evaluated against six different indicator bacteria in liquid mono-culture or co-culture, as shown in Figure 1a. We observed that the cell-free culture supernatant of Potato Dextrose Broth (PDB) (pH 7.0) exhibited inhibition on all tested strains, both in mono- and co-cultures. Interestingly, co-culture under PDB (pH 7.0) conditions improved the antibacterial activity against *B. subtilis* and *S. parasitica* with 36.67% and 42.86% improvement compared to pure monoculture, respectively. As the co-culture combination of *Streptomyces* sp. 2-85 and *Cladosporium* sp. 3-22 showed excellent effects, we chose the combination under PDB (pH 7.0) for further analysis.

In order to explore compounds potentially associated with antibacterial activity, we utilized LC-MS/MS under negative electrospray ionization (ESI^−^) conditions to cover as many compounds as possible in 2 mono-cultures and 4 co-cultures grown in different media (MM (Minimal Medium) or PDB) and pH conditions (pH 5.0 or 7.0). Unidentified molecular families may correspond to new molecules. A total of 1176 nodes were detected by the Mzmine software. Overall, approximately 43% (507 nodes) were unique to the mono-cultures, and about 12% (139 nodes) were newly synthesized during co-culture. Additionally, 45% (530 nodes) were shared between co-cultures and mono-cultures (Figure 1b). Among them, molecules detected during co-culture were mainly synthesized by fungal strains, possibly due to their larger proportion of the total biomass. In terms of different media, the total number of nodes produced in the PDB medium (532 nodes, 45%) was approximately twice that of the MM medium (286 nodes, 25%), with 26% of nodes showing an increase in new metabolites more than 4-fold (Figure 1c). It indicated that the PDB medium was significantly superior to the oligotrophic MM medium in metabolite production. Under different pH culture conditions, the total number of nodes produced at pH 7.0 (292 nodes, 25%) was approximately three times that at pH 5.0 (119 nodes, 10%), with 15% of nodes showing an increase in new metabolites more than 4-fold, too (Figure 1c). It suggested that the pH 7.0 culture condition was significantly better than pH 5.0 in terms of metabolite production. By comparing the structural spectra of compounds with the structures provided by MS-FINDER and determining the compound types based on matching mass peaks with MS/MS databases, only 13 specific compounds induced by co-culture were identified (Table S2), all of which were not detected in mono-cultures, indicating that the interactions between bacteria and fungi can activate or modulate biosynthetic pathways, thereby expanding the metabolite diversity.

### 2.3. Identification of Metabolites under Specific Cultivation Conditions (PDB, pH 7.0)

To unravel the mechanism behind the enhanced antibacterial effectiveness against *S. parasitica* through co-culture, we employed metabolomic analysis to identify metabolic variations between *Streptomyces* sp. 2-85 and *Cladosporium* sp. 3-22 when co-cultured in PDB medium at pH 7.0.

**Figure 2:**
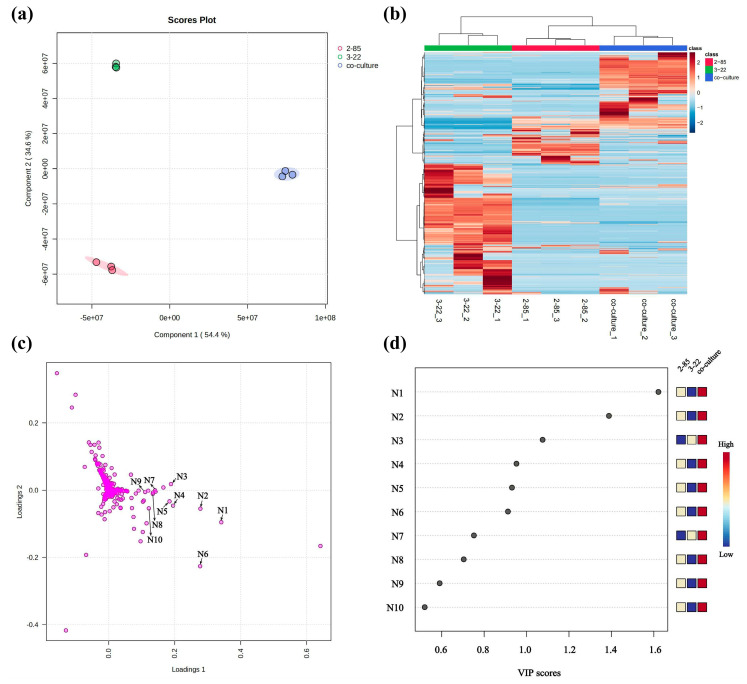
The PLS-DA (**a**), heatmap (**b**), loading plots (**c**), and VIP score (**d**) of co-culture of *Streptomyces* sp. 2-85 and *Cladosporium* sp. 3-22 and their corresponding mono-cultures. All the data were analyzed by LC-MS/MS in the positive mode. The scattered dots labeled with *m*/*z* values represented distinctive features among the three samples. These data were gathered from three separate biological replicates.

Partial least squares discriminant analysis (PLS-DA) was employed to analyze and visualize the metabolic features of co-cultured and mono-cultured samples under culture conditions of PDB medium at pH 7.0. The PLS-DA results indicated that the samples were divided into three groups, showing that the co-culture group did not overlap with the other two corresponding mono-culture groups, thus allowing for the differentiation of the chemical compositions between co-cultures and mono-cultures (Figure 2a). Hierarchical cluster analysis (HCA) of these 841 features based on MS data generated a heatmap, indicating global changes in the metabolome induced by co-culture (Figure 2b). Additionally, in the loading plot of PLS-DA, 69 compounds were off-center and clustered in the lower right corner of the plot (Figure 2c). Among them, 10 compounds (**N1**–**N10**) exhibited higher VIP scores in the PLS-DA analysis, indicating their significant contribution to the clusters (Figure 2d).

**Figure 3:**
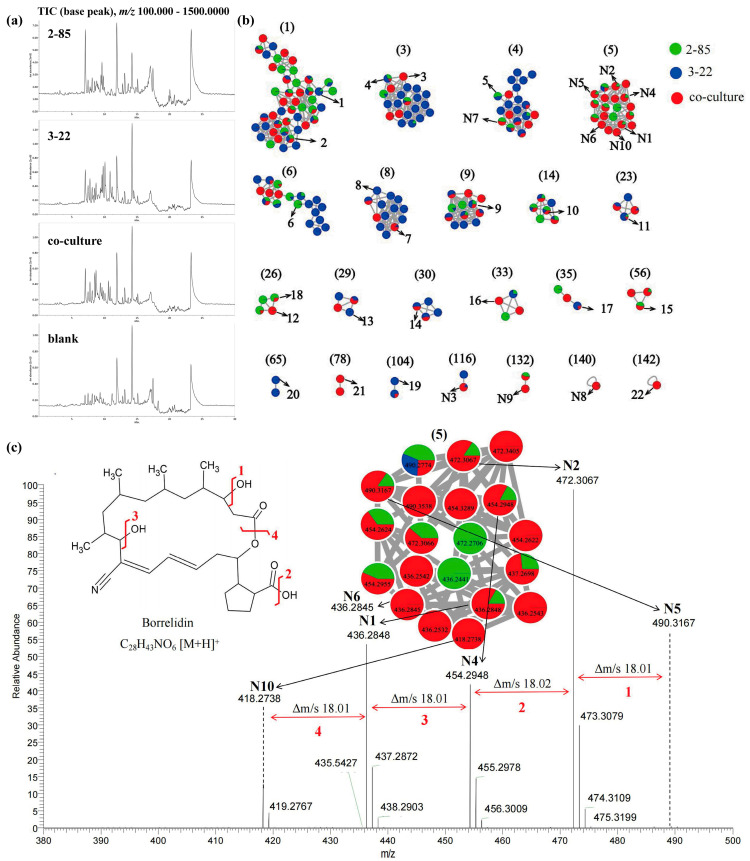
(**a**) Total ion chromatograms (+ ion mode) of EtOAc; (**b**) Global natural products social molecular networking (GNPS) analysis (+ ion mode) of compounds in the mono-*Streptomyces* sp. 2-85 and mono-*Cladosporium* sp. 3-22 and their co-cultures in PDB (pH 7.0); (**c**) Predicted MS/MS spectrum annotation of annotated borrelidin and its derivatives acquired by LC-MS/MS in positive ion mode.

To further explore the production of the most relevant active compounds potentially contributing to the observed antimicrobial activity, we analyzed the structures of the top 10 compounds identified by VIP. Among them, 6 priority molecules were detected at 14.93 min, namely N1 (*m*/*z* 436.2848 [M + H]^+^), N2 (*m*/*z* 472.3067 [M + H]^+^), N4 (*m*/*z* 454.2948 [M + H]^+^), N5 (*m*/*z* 490.3167 [M + H]^+^), N6 (*m*/*z* 436.2845 [M + H]^+^), and N10 (*m*/*z* 418.2738 [M + H]^+^). To more effectively visualize the differences in chemical profiles of strains under different culture conditions, the obtained data were transferred to the GNPS (Global Natural Products Social) molecular networks platform, where a Feature-Based Molecular Networking (FBMN) was generated. Subnetworks were exclusively generated from induced features lacking matches with database information. In each subnetwork, nodes represented parent ions and edges represented their structural relationships. It was found that these molecules clustered into one cluster in the molecular network (Figure 3c, cluster (5)), indicating close relationships among them. High-resolution mass spectrometry further confirmed that their molecular weights and fragmentation patterns were consistent with borrelidin. Among them, compound **N5** was the parent ion, while the others were dehydrated (-H_2_O) analogs of borrelidin. Borrelidin is an 18-membered macrolide polyketide originally isolated from *Streptomyces rochei*, with potent anti-microbial activity [18]. It is noteworthy that borrelidin is encoded by a unique BGC in the genome of *Streptomyces* sp. 2-85, further reinforcing this hypothesis.

Compared to the mono-culture extract of *Streptomyces* sp. 2-85, the content of borrelidin and its derivatives in the co-culture extract was significantly higher. The yields of compounds **N1, N2, N4,** and **N5** increased more than 4-fold, while compounds **N6** and **N10** also showed significant increases only in co-culture (Figure S2a). Considering the reported activity of borrelidin and the hypothetical annotation of its presence in the co-culture extract, we found that this extract exhibited strong activity against *S. parasitica*. Furthermore, the inhibitory activity of borrelidin increased with increasing concentration, indicating dose dependency, consistent with previous descriptions of its antimalarial activity [19]. This provides a potential explanation for the observed differences in activity induced by co-culture to some extent, suggesting that the enhanced anti-*S. parasitica* activity may be related to the significant increase in borrelidin production.

In order to clarify if other induced features corresponded to known metabolites, metabolite and chemical class annotations of 26 compounds within the other 21 distinct clusters were performed using the SIRIUS 4.9 workflow and manual dereplication based on comparative analysis of LC-MS/MS data in molecular networking (Figure 3a,b). Among them, 4 were exclusive to co-culture, 1 to *Streptomyces* sp. 2-85, and 4 to *Cladosporium* sp. 3-22, while 2 were shared between both mono-culture strains. High-resolution mass spectrometry confirmed that compounds **N3, N7, N8,** and **N9** with a relatively minor upregulation exhibited molecular weights and fragmentation patterns consistent with Okaramine, Tanzawaic acid F, Dysidazirine, and Antibiotic S-632-C, respectively, indicating diverse biological activities [20,21,22,23]. Compared to mono-cultures, compounds **3**, **12**, **16**, and **22** were only present in co-culture. Compound **21** exhibited the most significant increase, with a 30-fold increase in peak area. Compound **7** showed a 7-fold increase, while others increased slightly, covering various compound types (Figure S2b). Detailed annotations are provided in Table S3. Therefore, co-culture activation of silent biosynthetic pathways may amplify metabolic signatures, potentially uncovering novel molecules. This enhances active metabolite content, explaining the heightened antimicrobial efficacy observed in co-cultures compared to mono-cultures.

### 2.4. Isolation and Identification of Compounds

Considering that the putative borrelidin in the co-culture extract showed strong activity against *S. parasitica*, we isolated and purified this compound to evaluate its anti-*S. parasitica* activity and verify the putative annotation of this compound.

Compound **N5** was obtained as yellow amorphous powder and its molecular formula was assigned as C_28_H_42_NO_6_ by ESI-HRMS at *m*/*z* 489.3090 [M − H]^−^. Its specific rotation is [α]^25^_D_ − 0.41 (c 0.01, MeOH). Based on the comparison of the obtained MS (Figure S3), ^1^H-NMR, and ^13^C-NMR data (Figures S4 and S5, Table S4) with previously reported data, the structure of compound **N5** was determined to be borrelidin (Figure S6).

### 2.5. Composition and Functional Analysis of Compound ***N5***-Related Gene Clusters

**Figure 4:**
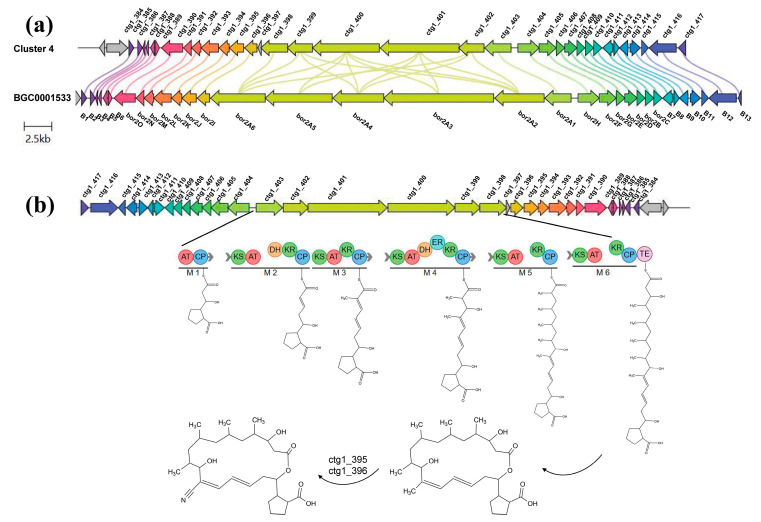
(**a**) Schematic diagram of comparison between the compound **N5**-related gene cluster and reference gene clusters. (**b**) The composition of the PKS module in the compound **N5**-related gene cluster and the proposed biosynthetic model.

Considering the chemical identification of compound **N5**, we found that the type I polyketide synthase (PKS) of hybrid cluster 4 shares 81% similarities to the BGC0001533 identified in *Streptomyces rochei* (GenBank: KT362046.1) (Figure 4a). Sequence analysis showed that this gene cluster has 36 distinct open reading frames (ORFs), all of which have the characteristic high G+C content typical of Streptomyces.

The gene responsible for the biosynthesis of compound **N5** spans approximately 52 kb, with six ORFs (ctg1_398 to ctg1_403) encoding a type I PKS gene cluster, consisting of 25 individual domains forming six modules. The protein sequences, products, and functions most similar to each ORF in the gene cluster were deduced through BLAST analysis (Table S5). Flanking the PKS genes are additional genes (ctg1_404 to ctg1_410 on the left side and ctg1_390 to ctg1_396 on the right side) also involved in compound **N5** biosynthesis. Some ORFs exhibit no significant similarity to known proteins, indicating they are not essential for compound **N5** biosynthesis.

Module 1 contains the AT and ACP domains. Each module contains conserved KS and ACP domains, along with AT domains exhibiting active site motifs containing serine residues. KR domains are present in all modules, with modules 2 and 4 also containing DH domains and module 4 additionally including an ER domain. Module 6 contains a thioesterase domain responsible for chain termination. Compound **N5** contains a nitrile group at C12 within its macrolide ring. Sequence analysis of the AT structures in each module suggests that the carbon atom in the nitrile group and the methyl in methylmalonyl-CoA are derived from the same source. The ctg1_396 gene encodes cytochrome P450 hydroxylase responsible for catalyzing the methylation at the C12 position, while ctg1_395 encodes an aminotransferase introducing the nitrogen atom into the activated C28 position. The ctg1_390 gene is presumed to encode a seryl-tRNA synthetase acting as a self-resistance gene. Upstream and downstream genes may be involved in biosynthesis initiation. For example, ctg1_391 encodes a protein similar to a key enzyme involved in the aromatic amino acid carboxylate isomerization, while ctg1_407 may participate in the cyclization process of intermediate products. Other genes like ctg1_409, ctg1_408, ctg1_394, and ctg1_392 encode enzymes with dual activities of dehydrogenase and reductase. The functions of ctg1_406, ctg1_405, ctg1_404, and ctg1_393 show no significant similarity to protein products obtained in the database and require further experimental validation. The composition and biosynthetic process of each PKS module are illustrated in Figure 4b.

### 2.6. Determination of EC_50_ for Inhibition of Mycelial Growth and Spore Germination of S. parasitica by Compound ***N5***

**Figure 5:**
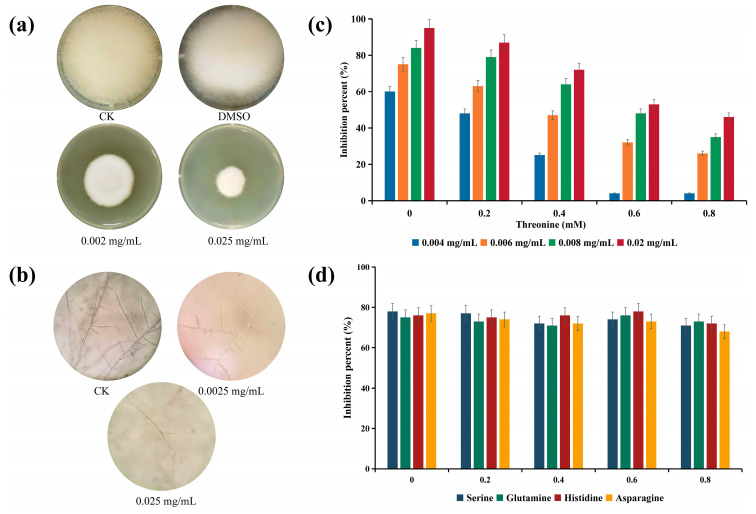
Inhibitory effects of compound **N5** on (**a**) the growth of mycelial and (**b**) spore germination of *S. parasitica*. (**c**) Effect of threonine on the inhibition activity of compound **N5** against *S. parasitica*. (**d**) Effect of serine, glutamine, histidine, and asparagine on the inhibition activity of compound **N5** against *S. parasitica*.

Compound **N5** displayed a 45% inhibition rate at a concentration of 0.002 mg/mL, which increased to 72.5% at 0.025 mg/mL (Figure 5a). Therefore, the compound exhibited a notable impact on restraining the mycelium growth of *S. parasitica* with an EC_50_ of 0.004 mg/mL.

Inhibition of zoospore germination contributes to reducing the rapid proliferation of propagules and is one of the important ways to mitigate diseases caused by *S. parasitica* [24]. Under different treatment concentrations, the inhibitory effect on zoospore germination can be distinctly observed through microscopy (Figure 5b). At a concentration of 0.0025 mg/mL of compound **N5**, spores germinated normally and formed branches, while at a concentration of 0.025 mg/mL of compound **N5**, germinating spores exhibited no apparent branching. At a concentration of 0.1 mg/mL of compound **N5**, zoospore germination was completely inhibited. Therefore, the EC_50_ for zoospore germination inhibition was determined to be 0.005 mg/mL.

### 2.7. Results of In Vitro Amino Acid Supplementation Experiments to Determine the Target of Action of Compound ***N5***

Previous studies have indicated that borrelidin exerts its inhibitory effect on *Phytophthora sojae* by inhibiting the activity of threonyl-tRNA synthetase, and this inhibitory effect is dose-dependent on the concentration of threonine [25]. Therefore, in this study, different concentrations of threonine were added to investigate whether the inhibitory effect of compound **N5** on *S. parasitica* shared the same target.

As shown in Figure 5c, with increasing concentrations of threonine, the inhibitory rate of compound **N5** on *S. parasitica* decreased, indicating a clear dose-dependent relationship between compound **N5** and threonine at different concentrations. When four other amino acids (serine, glutamine, histidine, and asparagine) were added to the culture medium, no similar dose-dependent relationship was observed (Figure 5d). Therefore, it can be preliminarily concluded that the target of compound **N5′**s inhibitory effect on *S. parasitica* is threonyl-tRNA synthetase.

## 3. Discussion

In this study, the genome of marine sponge-associated *Streptomyces* sp. 2-85 was found to harbor numerous unique BGCs, suggesting its potential to produce novel natural products. Co-cultivation with the fungus *Cladosporium* sp. 3-22 induced the production of 35 metabolites, including 19 exclusively observed in co-culture. Among them, borrelidin (compound **N5**) exhibited significant antifungal activity against *S. parasitica*, confirming its potential as a biocontrol agent. These results underscore the effectiveness of co-cultivation in generating new metabolites and the promising role of borrelidin in combating aquatic pathogens.

Streptomyces is known for its rich potential in natural product biosynthesis, typically harboring around 30 BGCs per genome [26]. *Streptomyces* sp. 2-85 stands out with 31 BGCs, indicating diverse secondary metabolite production, of which nearly 30% exhibited low similarities with annotated sequences, suggesting the presence of unknown metabolic pathways. Further exploration is crucial for uncovering novel bioactive compounds. The integration of genome mining, metabolomics, and co-culture of Streptomyces with other bacteria and fungi has been employed as an effective strategy to activate silent BGCs and discover novel bioactive secondary metabolites from marine sponges [27]. Competitive interactions between strains in co-cultures often enhance product yield or trigger the production of specific defensive metabolites. Recent studies have shown that Streptomyces-Fungi interactions can activate the BGCs of Streptomyces, yielding novel natural products [28]. In this study, compound **N5** and its derivatives, including compounds **N1, N2, N4, N6,** and **N10**, showed increased yields during co-culture, probably due to the mutual competition for nutrients or space. The Streptomyces-Fungi interaction also activated the borrelidin gene cluster, which might also trigger the expression of silent hydrolase genes, leading to lactone hydrolysis and the production of **N1, N2, N4, N6,** and **N10**. Similarly, unique fungal-bacterial interactions induce the production of fungal secondary metabolites, which have been confirmed at the molecular level. For example, the co-culture of *Aspergillus fumigatus* with the inducing strain *Streptomyces rapamycinicus* stimulated the production of fungal secondary metabolites fumicyclines A and B through histone modification [29]. The significant increase in fungal compound **21** production during co-culture may be related to such specific activation. Overall, Streptomyces serve as both inducers and participants in compound production during co-culture with fungi. In some cases, Streptomyces can trigger epigenetic modifications in fungi, resulting in complex secondary metabolic changes, while fungi can produce specific secondary metabolites that affect the secondary metabolism of Streptomyces.

Borrelidin was initially discovered in *Streptomyces rochei* isolated from soil samples and was found to exhibit anti-spirochetal activity [18]. Shi et al. first described the anti-oomycete activity of borrelidin against various *phytopathogenic oomycetes*, including *Pythium aphanidermatum*, *P. splendens*, *P. sylvaticum*, *P. ultimum*, and *P. capsica* [30]. Liu et al. also found borrelidin to display potent antibacterial activity against eight species of *Phytophthora sojae*, surpassing the commercial fungicide metalaxyl [31]. Despite borrelidin has previously shown antifungal and anti-oomycete activity, this study is the first to demonstrate its ability to inhibit the mycelial growth and spore germination of the aquatic pathogen *S. parasitica*. The compound targets threonyl-tRNA synthetase, consistent with previous studies suggesting its potential as a biocontrol agent for harmful aquatic pathogens [25]. Previous studies indicated that borrelidin is composed of nine ketone units [32], theoretically requiring eight extension steps, but the analysis of the composition and function of the gene cluster related to compound **N5** in this strain revealed only six extension modules, suggesting that two condensation steps containing malonyl-CoA units could not be identified. This may imply that the synthetic pathway of borrelidin may be more complex than previously expected or that other unknown synthetic pathways may exist in this strain to complete these missing steps.

In addition to highlighting the potent anti-*S. parasitic* activity of compound **N5**, this study also provides a series of candidate molecules for further investigation to identify other bioactive metabolites produced during the co-culture process. Optimum fermentation conditions were observed at PDB pH 7.0, inducing the production of metabolites, including sesquiterpenes, macrolides, polyketides, and amino acid derivatives, of which 9 metabolites were significantly increased. However, only borrelidin was detected in the extract, which was different from the predicted metabolites of the genome. This suggests that further optimization of co-culture conditions is needed to explore more compounds. We also detected the presence of borrelidin in the genome of *Streptomyces rochei* JK1 (CP121271.1), which shares high 16S rRNA gene homology (99.5%) with *Streptomyces* sp. 2-85, implying that this compound could be an important component of closely related strains.

## 4. Materials and Methods

### 4.1. Strains Isolation and Identification

*Streptomyces* sp. 2-85 (GenBank accession number PP784758) and *Cladosporium* sp. 3-22 (GenBank accession number PP818857) were isolated from marine sponges (Family Euretidae, Genus *Eurete*) collected in the South China Sea, China. The marine sponge was rinsed with sterile seawater and plated onto R2A (Reasoner’s 2A Agar, BD Difco, Becton Dickinson, MD, USA) and MEA (Malt Extract Agar, BD Difco, USA) agar medium at room temperature for isolation, upon which *Streptomyces* sp. 2-85 and *Cladosporium* sp. 3-22 were isolated separately. Pure cultures of each strain were preserved at −80 °C.

Genomic DNA was extracted using the Bacterial or Fungal Genomic DNA Extraction Kit (Qiagen, Hilden, Germany). 16S rRNA gene amplification using universal primers 27F and 1492R for bacteria and ITS1 (ITS1F 5′-CTTGGTCATTTAGAGGAAGTAA-3′; ITS2R 5′-GCTGCGTTCTTCATCGATGC-3′) for fungi (*Cladosporium* sp. 3-22 and *Saprolegnia parasitica* YG438). The cycling conditions for PCR amplification were optimized for each primer set as follows: 95 °C for 5 min, 30 cycles of 95 °C for 1 min, 55 °C for 1 min, 72 °C for 3 min, and a final extension of 72 °C for 5 min. The amplified PCR products were visualized by 1% agarose gel electrophoresis and then sequenced at Sangon Biotech (Shanghai, China). DNA sequences were edited and aligned with BioEdit 7.2 and subjected to the NCBI-BLAST tool (http://www.ncbi.nlm.nih.gov/BLAST/, accessed on 15 February 2024). The nucleotide accession number is available on GenBank. The reference strains of *Streptomyces* sp. 2-85 were chosen based on BLAST results, and the sequence similarity levels were determined through multiple sequence alignment using CLUSTAL W [33]. A neighbor-joining phylogenetic tree [34] was constructed with bootstrap values from 1000 replications [35] using MEGA version 11.0 (Arizona State University, Tempe, AZ, USA) [36].

### 4.2. Genome Mining and Identification of Compounds-Related Gene Custer

The nucleotide sequence data showed that *Streptomyces* sp. 2-85 (GenBank accession number PP784758) in this study shared the closest homology 99.8% with *Streptomyces rochei* S32 (GenBank accession number GCA 030908285.1) and the second closest homology 99.5% with *Streptomyces rochei* JK1 (GenBank accession number CP121271.1). The phylogenetic tree also indicates that they are evolutionarily closely related (Figure S1). Therefore, *Streptomyces rochei* S32 was chosen as the reference genome for subsequent experiments. Secondary metabolite biosynthetic gene clusters for *Streptomyces rochei* S32 were detected using antiSMASH 7.0 (https://antismash.secondarymetabolites.org/, accessed on 22 June 2024) with the default settings. The functional domain predictions for PKS/NRPS were analyzed using the PKS/NRPS Analysis Website (http://nrps.igs.umaryland.edu/, accessed on 20 March2024). The biosynthetic gene cluster sequences were selected for further analysis and annotation using BLAST (https://blast.ncbi.nlm.nih.gov/Blast.cgi, accessed on 15 February 2024).

### 4.3. Bioassays for Antibacterial and Antifungal Activity

We selected six indicator bacteria, including Gram-positive bacteria (*Staphylococcus aureus* ATCC6538, *Bacillus subtilis* ATCC6633, and *Geobacillus stearothermophilus* ATCC 7953), Gram-negative bacteria (*Escherichia coli* ATCC25922 and *Vibrio harveyi* 25919), and fungi (*Saprolegnia parasitica* YG438) for antimicrobial and antifungal activity tests. To prepare the pathogens or indicator microorganisms, *Staphylococcus aureus* ATCC6538, *Escherichia coli* ATCC25922, and *Bacillus subtilis* ATCC6633 were inoculated into 5 mL of LB (Luria–Bertani, Fisher Scientific, MA, USA) liquid medium, except *Vibrio harveyi* 25919 grown in MB liquid medium (marine broth 2216, BD Difco, USA) and *Geobacillus stearothermophilus* ATCC 7953 cultivated in TSB (tryptic soy broth, BD Difco, USA) liquid medium. The inoculated flasks were placed in an incubator at 37 °C and 180 rpm for 12 h for *S. aureus*, *E. coli*, and *B. subtilis*, at 28 °C and 180 rpm for 12 h for *V. harveyi*, and at 55 °C and 180 rpm for 24 h for *G. stearothermophilus*, until OD_600_ reached 0.4, respectively. Each solution of pathogenic bacteria is mixed with LB or MB agar media at a volume ratio of 1:100 to make the test plates. Sterilized filter paper disks (Difco, 6 mm in diameter) were arranged on the plate, and 10 μL of each sample was added to the paper disks, incubating at 37 °C, 28 °C, or 55 °C for 24 h, after which the diameters of the inhibition zone were measured.

*S. parasitica* YG438 (fish isolate, GenBank accession number PP814841) was inoculated onto PDA (Potato Dextrose Agar, 20 g of glucose, 4 g of potato infusion, 30.0 g of sea salts, and 7.5 g of Bacto Agar in 1-L dH_2_O) solid medium and incubated at 28 °C for 5 days. The spore suspension was prepared by washing twice with sterile saline and counted using a blood counting chamber, with the final concentration of 1.0 × 10^7^ CFU/mL. The antifungal activity of the supernatant was tested by the filter paper disc diffusion method as described above. That is, a disc with 10 μL supernatant (6 mm) of each strain was positioned in the middle of the fungus-inoculated plates (spore suspension mixed with PDA agar media, 1:100 *v*/*v*) and incubated for 24 h at 28 °C.

### 4.4. Strains Cultivation and Co-Culture Conditions

*Streptomyces* sp. 2-85 and *Cladosporium* sp. 3-22 were initially activated on solid ISP2 (4.0 g of yeast extract, 10.0 g of malt extract, 4.0 g of glucose, and 30.0 g of sea salts in 1-L dH_2_O) or PDA media at 28 °C for 7 d, respectively. Subsequently, *Streptomyces* sp. 2-85 was inoculated into 50 mL of ISP2 liquid medium and incubated at 28 °C at 180 rpm/min until reaching an optical density (OD) of OD_600_ = 0.5, *Cladosporium* sp. 3-22 was grown on PDA solid medium at 28 °C to obtain an appropriate concentration of conidia (1 × 10^6^ CFU/mL), serving as the respective liquid seed culture.

A liquid co-culture system with different media and pH (pH 5.0 or 7.0) of *Streptomyces* sp. 2-85 and *Cladosporium* sp. 3-22 was established. Briefly, for pre-culture, the pre-cultured *Streptomyces* sp. 2-85 and *Cladosporium* sp. 3-22 were added to 50 mL of MM (Minimal Medium, 1.98 g of ammonium sulfate, 0.68 g of potassium dihydrogen phosphate, 0.20 g of magnesium sulfate heptahydrate, 0.011 g of ferric sulfate, 4.55 g of mannitol, 11.05 g of N-acetylglucosamine, and 20.0 g of sea salts in 1-L dH_2_O) or PDB (Potato Dextrose Broth, 20 g of glucose, 4 g of potato infusion, and 30.0 g of sea salts in 1-L dH_2_O) media in the same proportion and incubated at 28 °C for 3 days by shaking at 180 rpm/min. Then, the co-preculture was inoculated in 100 mL of MM or PDB (pH 5.0 or pH 7.0) liquid media at a volume ratio of 1:100 and cultured at 28 °C on a rotary shaker at 180 rpm/min for 10 days. For pure culture, *Streptomyces* sp. 2-85 and *Cladosporium* sp. 3-22 were grown in 100 mL MM or PDB (pH 5.0 or pH 7.0) liquid media at 28 °C for 10 days by shaking at 180 rpm/min, respectively.

### 4.5. Extraction of Mono- and Co-Cultures

Each culture was homogenized with ethyl acetate (EtOAc, 1:1 *v*/*v*, Sigma-Aldrich, St. Louis, MO, USA) using Ultra-Turrax (Miccra, Mülheim, Germany) at 13,000 rpm for 30 s and left overnight on a rotary shaker at 120 rpm. An additional 100 mL of ethyl acetate was added, and the mixture was sonicated for 5 min and stirred for 10 min. This step was repeated twice. The three extracts of each sample were combined and the supernatant was washed with an equal volume of Milli-Q water (Arium^®^ Lab water systems, Sartorius, Göttingen, Germany) to remove media components. The organic extracts were combined and dried using a rotary evaporator (Rotavapor RE120; Büchi Labortechnik, Flawil, Switzerland) at 39 °C. Finally, the extract of each sample was weighed and then dissolved in methanol (MeOH, Sigma-Aldrich, St. Louis, MO, USA) to obtain a final concentration of 100 μg/μL. All extracts were stored at −20 °C for further use.

### 4.6. LC-MS/MS Analysis

Chromatographic analysis was performed on an UltiMate^TM^ 3000 HPLC system (Thermo Fisher Scientific, Cleveland, OH, USA) interfaced with a Q-Exactive quadrupole orbitrap mass spectrometer (Thermo Fisher Scientific), using a heated electro-spray ionization (HESI-II) source. All extracts were prepared for LC-MS/MS at 0.1 mg/mL in HPLC MeOH. 5 µL of each sample were injected onto a ZORBAX SB-C_18_ column (4.6 mm × 250 mm, 5 µm; Agilent, Pal Alto, CA, USA) with a flow rate of 0.4 mL/min, operating at 25 °C. The gradient procedure was as follows: (A) MilliQ-water with 0.1% formic acid (UPLC/MS grade); (B) acetonitrile (MeCN, UPLC/MS grade, Sigma-Aldrich, St. Louis, USA); 5–95% B (0–15.0 min); 95% B (15.0–20.0 min), 95–5% B (20.0–20.1 min), 5% B (20.1–30.0 min). Optimized HESI-II parameters were as follows: source voltage: +4.0 kV (positive ion mode), −3.5 kV (negative ion mode); cone voltage: 60.0; capillary temperature: 350 °C; curtain gas, ion source gas 1, and ion source gas 2 were set at 35, 50, and 50 psi; full-scan acquisition in the mass range of *m*/*z* 100 to 1500; and MS2 fragmentation was obtained with a low ramp collision energy (CE) of 6–60 eV and high CE of 9–80 eV.

### 4.7. Metabolite Profile and Structural Analysis

To fully understand the differences in metabolic profiles between co-culture and mono-culture, multiple tools such as MS-DIAL 4.0 (The University of Tokyo, Tokyo, Japan) [37], MS-FINDER 3.52 (Kyoto University, Kyoto, Japan) [38], MetaboAnalyst 6.0 (University of Alberta, Edmonton, AB, Canada) [39], and GNPS (Global Natural Products Social) molecular networking [14] were integrated for compound analysis and identification in the extracts. The method mainly includes: Using MS-DIAL 4.0 to determine the monoisotopic mass of peaks and define adduct ions as [M + H]^+^, [M + K]^+^, [M-H_2_O + H]^+^, [M + Na]^+^, and [2M + H]^+^ (positive ion mode), [M − H]^−^, [M − H_2_O − H]^−^, [M + C_2_H_3_N − H]^−^, [M + CH_2_O_2_ − H]^−^, [M + C_2_H_4_O_2_ − H]^−^, and [2M − H]^−^ (negative ion mode). When at least two adduct ions match the adduct ion dictionary, the monoisotopic mass of each peak is determined. The mass signal extraction range was set to 100–1500 Da with a mass tolerance of 0.25 D. Metabolite ions were converted into structural information by MS-FINDER 3.52, and then compared with public spectra in MS/MS databases, including NIST 14 (NIST14, version 2.2, National Institute of Standards and Technology, Gaithersburg, MD, USA), Metlin [40], ReSpect [41], MassBank [42], and MetaboBase [43]. Estimated compound MS2 fragments were compared with the structures of corresponding known compounds using SIRIUS 4.9 (Max Planck Institute of Biophysics, Frankfurt, Germany) [44] and CFM-ID 4.0 (University of Toronto, Toronto, ON, Canada) [45]. Compounds with monoisotopic mass errors within ±5 ppm and structure score higher than 5 were selected for mass spectral peak matching and searched in the SciFinder database to confirm whether they are natural products [46]. Subsequently, MetaboAnalyst 6.0 (University of Alberta, Edmonton, Canada) was used for multivariate analysis of the overall metabolite spectrum [39]. The aligned data were normalized and then analyzed using PLS-DA (Partial Least Squares Discriminant Analysis) to reveal global metabolic changes and obtain heatmaps that can display feature clustering and visualize intergroup differences. Finally, the mass spectrometry data were uploaded to GNPS (Global Natural Products Social) to create a network of metabolites for analyzing relationships between compound structures. The mass spectrometry data were first converted to mzXML format by MSConvert software (ProteoWizard 3.0, Vanderbilt University, Nashville, TN, USA) and processed by Mzmine 2.53 before being uploaded to GNPS (Global Natural Products Social) [47]. The FBMN (Feature-Based Molecular Networking) workflow was applied for analysis, generating molecular networks with the following parameters: precursor ion mass tolerance of 0.05 Da, MS/MS fragment ion tolerance of 0.05 Da, cosine score > 0.7, minimum number of matching peaks ≥ 6, and maximum number of neighboring nodes = 10. Finally, the molecular network was visualized using Cytoscape 3.9.1 software (University of California, San Diego, CA, USA) [48], where nodes in the molecular network represented molecules and the edges between nodes represented the size of interactions between molecules. Blank culture media and solvent methanol from the control group were excluded.

### 4.8. Inhibitory Effects of Isolated Compound on Mycelial Growth and Spore Germination of S. parasitica

Different concentrations (0.002, 0.005, 0.008, 0.01, 0.02, 0.04, and 0.05 mg/mL) of the isolated compound were mixed with molten PDA medium to assess their EC_50_ against *S. parasitica.* After solidifying the agar plates, a 6-mm mycelial plug of *S. parasitica* was carefully placed at the center of each treated agar plate and incubated in the dark for 24 h at 28 °C. Using metalaxyl as a positive control and DMSO as a negative control. The mycelial growth inhibition rate (%) was determined using the formula (1 − d1/d2) × 100, where d1 and d2 are the mean colony diameters of the test samples and the mean colony diameters of the control, respectively. The dose-response curve was generated to determine the effective concentration (EC_50_) using GraphPad Prism 10.0 (GraphPad Software, La Jolla, CA, USA).

In addition, the EC_50_ values were determined to assess the inhibitory effect of isolated compounds on the spore germination of *S. parasitica*. Each well of the 96-well plate was filled with 50 μL of both PDB medium and spore suspension of *S. parasitica* (~100 CFU/mL), followed by the addition of extracts at final concentrations of 0.0025, 0.005, 0.01, 0.025, 0.05, and 0.1 mg/mL, using metalaxyl as a positive control and DMSO as a negative control. After incubating the mixtures for 24 h at 28 °C, the EC_50_ was estimated based on microscopic observation of spore germination and growth inhibition, with the length of the germ tube not exceeding half of the spore diameter.

### 4.9. Compound Isolation

The co-incubation experiment was performed to track compounds. Briefly, *G. stearothermophilus* (10^8^ CFU/mL) was centrifuged at 8000× *g* for 3 min. Bacterial cells were retained and excluded from the medium by rinsing 3 times using a sterile PBS buffer. Incubated with crude extract of co-cultures of *Streptomyces* sp. 2-85 and *Cladosporium* sp. 3-22 (PDB, pH 7.0) for 1 h at 37 °C. The suspension was obtained by centrifugation at 3000× *g* for 10 min and filtered through a 0.2 µm filter. Test samples, with a final concentration of 0.1 mg/mL, were analyzed by HPLC using a ZORBAX SB-C_18_ column (4.6 mm × 250 mm, 5 µm; Agilent, USA) at a flow rate of 0.4 mL/min and operating at 25 °C. Gradient elution was employed with the following parameters: (A) water with 0.1% ammonium acetate; (B) acetonitrile; 5–95% B (0–20.0 min); 95% B (20.0–30.0 min), 95–5% B (30.0–30.1 min), 5% B (30.1–40.0 min). DMSO was used as a control, and samples without extract served as negative controls.

The co-culture of *Streptomyces* sp. 2-85 and *Cladosporium* sp. 3-22 (PDB, pH7.0) (2 L) was extracted with EtOAc, yielding a crude extract (446.6 mg) following the same extraction steps as described above. The purification of the antimicrobial compound was initially carried out using semi-preparative HPLC with an Agilent 1100 series (G1322A degasser, G1311A quaternary pump with a G1363A extended volume upgrade kit, G1313A autosampler, G1316A column thermostat, and G1364 fraction collector; Agilent Technologies Inc., Palo Alto, CA, USA). Separation was achieved on an Agilent ZORBAX SB-C_18_ column (10 × 250 mm, 5 µm). The mobile phase consisted of a mixture of (A) water with 0.1% ammonium acetate (*v*/*v*) and (B) acetonitrile and was pumped at a rate of 3 mL/min. 100 μL of sample solution (5 mg/mL) was injected. The gradient elution program was from 5% B to 95% B to yield 4 fractions. Antimicrobial activity was checked, and the most active fraction (fraction A) was used for further analysis. Fraction A was further purified using an Agilent HPLC system using an Agilent ZORBAX SB-C_18_ column (4.6 × 250 mm, 5 µm) with gradient elution as follows: (A) water with 0.1% ammonium acetate; (B) acetonitrile; 5–40% B (0–5.0 min); 40–46% (5.1–45.0 min); 46–95% B (45.1–55.0 min); 95–5% B (55.1–56.0 min); 5% B (56.1–60.0 min) with a 1 mL/min flow led to the isolation of 16 mg of compound **N5**.

## Data Availability

The mass spectrometry data are available under the MassIVE ID (MSV000094797) and MassIVE ID (MSV000094798). The molecular networking job is available online at https://gnps.ucsd.edu/ProteoSAFe/status.jsp?task=a020b29ce4b04309928bd6b8b8f2af3f (accessed on 14 April 2024) and https://gnps.ucsd.edu/ProteoSAFe/status.jsp?task=6694e8d29a7e43c5bcdce6217867219e (accessed on 27 March 2024).

## Supplementary Materials

The following supporting information can be downloaded at: https://www.mdpi.com/article/10.3390/md22070302/s1, Figure S1: Phylogenetic analysis of *Streptomyces* sp. 2-85 based on the Neighbor-Joining (NJ) method. Bootstrap values based on 1000 replicates are shown at tree nodes. The scale bar at the bottom of the tree indicates K2P genetic distance; Figure S2: (a,b) The changes in the peak area (AUC) of annotated compounds of *Streptomyces* sp. 2-85 and *Cladosporium* sp. 3-22 under co-culture conditions; Figure S3: ESI-HRMS mass spectra of compound N5 in negative ion mode; Figure S4: 1H NMR spectrum of compound N5 (600 MHz, CDOD3); Figure S5: 13C NMR spectrum of compound N5 (600 MHz, CDOD3); Figure S6: Structure of compound N5. Table S1: Inferred BGCs in *Streptomyces* sp. 2-85 based on antiSMASH 7.0 analysis of *Streptomyces* rochei S32 genome; Table S2: Putatively identified compounds from co-culture strains of *Streptomyces* sp. 2-85 and *Cladosporium* sp. 3-22 using co-cultivate method (-ion mode); Table S3: Putatively identified compounds from mono- or co-culture strains of *Streptomyces* sp. 2-85 and *Cladosporium* sp. 3-22 under PDB, pH7.0 condition (+ ion mode); Table S4: 1H (600 MHz) and 13C (125 MHz) NMR data in CDOD3; Table S5: Putative functions for genes in the compound **N5**-related gene cluster.
