# Supplementary material for: Streptomyces-Fungus Co-Culture Enhances the Production of Borrelidin and Analogs: A Genomic and Metabolomic Approach
Source: Mar Drugs. 2024 Jun 28;22(7):302. doi: 10.3390/md22070302 (PMC11278061; doi:10.3390/md22070302)
Supplement: Supplementary file 1 [file marinedrugs-22-00302-s001.zip › marinedrugs-3046526-supplementary.pdf]

Supporting information

Streptomyces-Fungus Co-Culture Enhances the Production of Borrelidin and Analogs: A Genomic and Metabolomic Approach

Tan Liu [1], Xi Gui [1], Gang Zhang [2], Lianzhong Luo [2] and Jing Zhao [1,*]

[1]*College of Ocean and Earth Science, Xiamen University, Xiamen 361005, China*
[2]*Xiamen Key Laboratory of Marine Medicinal Natural Product Resources, Xiamen Medical College, Xiamen 361005, China*

Content

Table S1. Inferred BGCs in *Streptomyces* sp. 2-85 based on antiSMASH 7.0 analysis of *Streptomyces rochei* S32 genome

Table S2. Putatively identified compounds from co-culture strains of *Streptomyces* sp. 2-85 and *Cladosporium* sp. 3-22 using co-cultivate method (- ion mode)

Table S3. Putatively identified compounds from mono- or co-culture strains of *Streptomyces* sp. 2-85 and *Cladosporium* sp. 3-22 under PDB, pH7.0 condition (+ ion mode)

Table S4. ^{1}H (600 MHz) and ^{13}C (125 MHz) NMR data in $CDOD_3$

Table S5. Putative functions for genes in the compound **N5**-related gene cluster

Figure S1. Phylogenetic analysis of *Streptomyces* sp. 2-85 based on the Neighbor-Joining (NJ) method. Bootstrap values based on 1000 replicates are shown at tree nodes. The scale bar at the bottom of the tree indicates K2P genetic distance.

Figure S2. (a, b) The changes in the peak area (AUC) of annotated compounds of strain *Streptomyces* sp. 2-85 and *Cladosporium* sp. 3-22 under co-culture conditions

Figure S3. ESI-HRMS mass spectra of compound **N5** in negative ion mode

Figure S4. ^{1}H NMR spectrum of compound **N5** (600 MHz, $CDOD_3$)

Figure S5. ^{13}C NMR spectrum of compound **N5** (600 MHz, $CDOD_3$)

Figure S6. Structure of compound **N5**

Table S1. Inferred BGCs in *Streptomyces* sp. 2-85 based on antiSMASH 7.0 analysis of *Streptomyces rochei* S32 genome

Cluster	Type	Length (bp)	Most similar known cluster	Similarity
1	T2PKS,butyrolactone	85357	fluostatins M-Q	65%
2	lanthipeptide-class-iii	21502	SAL-2242	88%
3	lanthipeptide-class-i	24606		
4	T1PKS,NRP-metallop hore,NRPS	107042	borrelidin	81%
5	RiPP-like	10215	informatipeptin	42%
6	terpene	21031		
7	T1PKS	94850	desertomycin B/desertomycin A/desertomycin G	30%
8	hydrogen-cyanide	12870	aborycin	28%
9	terpene	26719	hopene	100%
10	NRPS,arylpolyene	90300	lipopeptide 8D1-1/lipopeptide 8D1-2	84%
11	NI-siderophore	29499	paulomycin	13%
12	terpene	22180	geosmin	100%
13	RiPP-like	11331		
14	NI-siderophore	29877	kinamycin	16%
15	T2PKS	72596	spore pigment	66%
16	terpene	21091	albaflavenone	100%
17	lanthipeptide-class-iii	22294	catenulipeptin	40%
18	lanthipeptide-class-v	42323	pristinin A3	11%
19	PKS-like,furan	40956	methylenomycin A	9%
20	NI-siderophore	29772	desferrioxamin B/desferrioxamine E	100%
21	melanin	10608	istamycin	4%
22	ectoine	10398	ectoine	100%

23	T3PKS	41106	flaviolin/1,3,6,8-tetra hydroxynaphthalene	100%
24	NRPS-like	42006	streptothricin	95%
25	terpene	34875	isorenieratene	63%
26	indole	21121	5-dimethylallylindol e-3-acetonitrile	100%
27	terpene	21691	lysolipin I	4%
28	hglE-KS,T1PKS,NRPS-like,NRPS	208122	candicidin	95%
29	terpene	25581	isorenieratene	100%
30	indole	21178	7-prenylisatin	83%
31	T2PKS,butyrolactone	65167	fluostatins M-Q	44%

Table S2. Putatively identified compounds from co-culture strains of *Streptomyces* sp. 2-85 and *Cladosporium* sp. 3-22 using co-cultivate method (- ion mode)

Comp. No.	tR (min)	Precursor m/z	chemical compound family	Molecular formula (Δ ppm)	Cultivation Type	Classification	MS/MS fragments	Biological source
1	12.74	346.1564 $[M-H]^-$	CID 122209644	$C_{18}H_{23}N_2O_5$ (-0.011)	MM, pH 7.0	—	260.1674；204.1030；164.0700；124.0390；	—
2	13.61	322.1642 $[M-H]^-$	Antibiotic S 632-B1	$C_{17}H_{25}NO_5$ (0.968)	PDB, pH 5.0 or 7.0	Glutarimide antibiotic	254.1781；212.1658；	*Streptomyces hygroscopicus* S-632
3	14.01	524.2998 $[M+C_2H_3N-H]^-$	Farneside B	$C_{24}H_{40}N_2O_8$ (-1.322)	MM, pH 5.0	Sesquiterpenoid	426.3008；271.1918；599.0122；	*Streptomyces* sp. CNT-372
4	13.86	368.1485 $[M+CH_2O_2-H]^-$	CID 101850356	$C_{17}H_{23}O_6$ (1.469)	PDB, pH 5.0 or 7.0	—	324.1566；280.1699；91.7971；	—

5	11.36	502.1705 [M+CH2O2-H]$^-$	Medermycin	$C_{24}H_{27}NO_8$ (0.368)	MM, pH 5.0	Benzo isochroman quinone	434.1822；337.9365；181.0873；91.7988；	*Streptomyces* sp. XMA39
6	13.05	635.3332 [M-H]$^-$	UCF-116-B	$C_{36}H_{48}N_2O_8$ (-2.16)	MM, pH 7.0	N-acyl-amino acid	500.2662；435.2312；318.1727；247.1346；148.0395；134.0601；	*Streptomyces* sp.
7	14.63	377.1734 [M-H]$^-$	Antibiotic Bu2545	$C_{16}H_{30}N_{20}O_6S$ (1.001)	MM, pH 7.0	Glutarimide antibiotic	333.1493；209.0602；144.0729；91.7979；	*Streptomyces* sp. No. H230-5
8	14.76	368.2056 [M-H]$^-$	Cinnabaramide D	$C_{19}H_{31}NO_6$ (-2.941)	PDB, pH 7.0	Alpha-amino acid	232.0974；112.9845；	*Streptomycete* sp.
9	13.83	408.2106 [M+C2H3N-H]$^-$	Glycylhistidylarginine	$C_{14}H_{24}N_8O_4$ (-1.156)	MM, pH 7.0	Oligopeptide	317.1260；283.1338；239.1440；226.0879；181.0849；134.0232；124.0389；91.0540；	—
10	14.16	534.3447 [M-H2O-H]$^-$	JuVenimicin A2	$C_{30}H_{51}NO_8$ (2.932)	MM，PDB, pH 5.0 or 7.0	Aminoglycoside	490.3516；472.3407；412.3252；353.2860；	*Micromonospora* sp.

							218.1532；59.0122；	
11	14.93	488.3390 [M-H]$^-$	Borrelidin	$C_{28}H_{43}NO_6$ (3.013)	MM, PDB, pH 5.0 or 7.0	Polyketide	426.3040；271.1923；172.1124；59.0124；	*Streptomyces coelicoflavus*
12	9.67	313.1200 [M+$C_2H_4O_2$-H]$^-$	Oxopropaline G	$C_{15}H_{14}N_2O_2$ (0.119)	PDB, pH 5.0 or 7.0	Alkaloid	285.1244；269.1279；251.1185；241.1341；122.0605；	*Streptomyces* sp.
13	13.98	588.3147 [M-H]$^-$	KRN-5500	$C_{28}H_{43}N_7O_7$ (-0.577)	MM, pH 5.0 or 7.0	Nucleoside-like antineoplastic antibiotic	475.5833；314.9197；213.1336；116.9266；91.7971；	*Streptomyces alanosinicus*

Table S3. Putatively identified compounds from mono- or co-culture strains of *Streptomyces* sp. 2-85 and *Cladosporium* sp. 3-22 under PDB, pH7.0 condition (+ ion mode)

No.	RT (min)	Observed m/z	Calculated (m/z)	Δppm	Molecular formula	Compounds' name	Classification	Cultivation Types	MS/MS fragments
N1	14.93	436.2848 $[M+H]^+$	435.6080	2.669	$C_{28}H_{37}NO_3$	Borrelidin's derivatives	Polyketide	2-85, co-culture	418.2735；390.2806；303.1225；238.1211；21.1076；147.0796；123.0812；95.0863
N2	14.93	472.3067 $[M+H]^+$	471.6380	1.460	$C_{28}H_{41}NO_5$	Borrelidin's derivatives	Polyketide	2-85, co-culture	454.2914；436.2819；418.2735；408.2892；390.2831；238.1211；184.1114；123.0812
N3	7.77	561.2519 $[M+Na]^+$	538.2580	1.463	$C_{32}H_{34}N_4O_4$	Okaramine	Indoles	3-22, co-culture	455.1861；411.1601；385.1463；309.1017；293.1090；269.0715；241.0759
N4	14.93	454.2948 $[M+H]^+$	453.6230	3.785	$C_{28}H_{39}NO_4$	Borrelidin's derivatives	Polyketide	2-85, co-culture	418.2735；408.2881；390.2806；238.1211；184.1118；123.0810；95.0864
N5	14.93	490.3167 $[M+H]^+$	489.6452	1.982	$C_{28}H_{43}NO_6$	Borrelidin	Polyketide	2-85, co-culture	436.2819；418.2735；408.2881；390.2806；238.1211；184.1118；172.1127；147.0796；123.0814
N6	14.93	436.2845 $[M+H]^+$	435.6080	3.458	$C_{28}H_{37}NO_3$	Borrelidin's derivatives	Polyketide	co-culture	418.2735；390.2806；303.1225；238.1211；147.0796；123.0812

N7	7.75	327.1556 [M+Na]+	304.1675	1.664	$C_{18}H_{24}O_4$	Tanzawaic acid F	Terpenoid	2-85, co-culture	234.1116；207.1128；184.0595；166.0511
N8	10.75	346.2170 [M+K]+	307.2511	3.641	$C_{19}H_{33}NO_2$	Dysidazirine	Alpha-amino acid ester	2-85, co-culture	301.1939；221.1299；207.1162；169.1006；155.0863；129.0696；105.0707；91.0545
N9	13.94	330.1704 [M+Na]+	307.1784	2.495	$C_{17}H_{25}NO_4$	Antibiotic S-632-C	Lactone	2-85, co-culture	270.1469;98.9843;91.8134
N10	14.93	418.2738 [M+H]+	417.5930	1.328	$C_{28}H_{35}NO_2$	Borrelidin's derivatives	Polyketide	co-culture	165.1072；147.0813；123.0812；120.0886；91.8142
1	13.26	390.2854 [M+H]+	389.2777	2.178	$C_{20}H_{39}NO_6$	sphingofungin B	Long-chain fatty acids	2-85, 3-22	328.3213；284.2965；149.0243；132.1017；91.8167
2	12.53	318.0917 [M+H]+	317.0859	3.481	$C_{11}H_{15}N_3O_8$	Polyoxin C	Polypeptides	2-85, 3-22, co-culture	256.2628；102.0919；91.8135；88.0759；70.0656
3	10.90	333.2048 [M+H]+	332.1988	1.493	$C_{20}H_{28}O_4$	Kericembrenolide	Terpenoids	co-culture	271.2072；189.1270；175.1116；159.1169；135.1163；109.1020
4	10.91	333.2057 [M+H]+	332.1988	1.295	$C_{20}H_{28}O_4$	Sinularolide E	Macrolides	2-85, 3-22,	271.2072；189.1270；175.1116；161.0963；159.1169；135.1163；109.1020

								co-culture	
5	8.37	413.2661 [M+H]+	412.2573	-2.688	$C_{21}H_{36}N_2O_6$	Epopromycin A	carbonyl compounds	2-85	234.1113;208.0980;184.0593;166.0510;162.0664;147.0440
6	10.89	311.1968 [M+H]+	310.1780	3.208	$C_{17}H_{26}O_5$	Oxycineromycin B	Macrolides	2-85,3-22	131.0848；125.0964；109.1020；95.0863；81.0706；79.0545；67.0552；55.0552
7	12.36	392.2073 [M+Na]+	369.2151	3.067	$C_{19}H_{31}NO_6$	cinnabaramide D	Alpha-amino acid	3-22, co-culture	314.1739；286.1798；272.1630；234.1117；220.1321；192.1026；152.0715；149.0227
8	9.86	332.1125 [M+Na]+	309.1212	3.617	$C_{15}H_{19}NO_6$	Sporovexin C	Alpha-amino acid	3-22	220.1350；192.1026；175.1116；152.0715；98.9843
9	14.86	460.3033 [M+H]+	459.2985	2.406	$C_{27}H_{41}NO_5$	Piericidin C3	Sesquiterpenoids	2-85, 3-22, co-culture	135.0805；119.0853；107.0867
10	14.87	446.2538 [M+H]+	445.2464	3.492	$C_{25}H_{35}NO_6$	Metachromin N	Prenol lipids	2-85, 3-22, co-culture	357.6964；118.0506；100.0400；72.0450；62.0605
11	14.64	373.1999 [M+Na]+	350.2093	2.218	$C_{20}H_{30}O_5$	Phomactin M	Alcohols and polyols	2-85, 3-22,	313.2144；295.2051；271.2029；245.1539；211.1497；

								co-culture	175.1115；157.1009；95.0863
12	14.20	518.3268 [M+H]+	517.3192	1.606	$C_{33}H_{43}NO_4$	Sarcotrine B	Terpenoids	co-culture	392.2776；301.2137；245.1540；227.1422；199.1482；171.1168；159.1169；121.1015
13	10.05	385.0908 [M+Na]+	362.1002	1.569	$C_{18}H_{18}O_8$	Methyl Asterrate	Diphenylethers	3-22	299.1121；281.1000；213.0720；195.0637
14	11.31	331.0967 [M+K]+	292.1311	2.669	$C_{16}H_{20}O_5$	Asperentin	Benzenoids	3-22, co-culture	174.2967；160.7361；121.1015；98.9843；91.8125
15	14.02	408.2905 [M+H]+	407.2824	-1.475	$C_{27}H_{37}NO_2$	GT32-B	Macrolactams	2-85, co-culture	408.2881；263.5277；210.1289；184.1120；172.1127；123.0799；91.8134
16	7.00	328.2233 [M+H]+	327.2158	3.128	$C_{16}H_{29}N_3O_4$	Diprotin B	Tripeptide	co-culture	305.1295；287.1194；285.1029；269.1053
17	13.47	455.2581 [M+Na]+	432.2696	3.006	$C_{18}H_{36}N_6O_6$	Istamycin A2	Aminocyclitol Glycosides	3-22, co-culture	399.2456；239.5356；158.0805；116.0706；91.8159；70.0661；60.0815
18	10.97	494.3488 [M+H]+	493.3403	1.371	$C_{28}H_{47}NO_6$	Holantosine E	Steroids	2-85, co-culture	476.3362；336.2505；245.1542；227.1424；199.1484；147.1170；119.0855；107.0862；95.0864
19	13.70	465.3802	464.3767	3.031	$C_{31}H_{48}N_2O$	Plakinamine H	Triterpenoids	3-22	401.1441；267.1519；219.1500；191.1188；

		[M+H]+							120.0815；91.8142；72.0814
20	11.64	461.2408 [M+K]+	422.2821	3.228	$C_{28}H_{38}O_3$	Minabeolide-1	Steroids	3-22	309.2200；295.2054；269.1887；251.1797；241.1970；175.1111；147.1168；133.1009；109.1020；95.0865
21	7.27	329.0722 [M+H]+	328.0583	0.913	$C_{17}H_{12}O_7$	Aflatoxin G1	Furanocounmarins	3-22, co-culture	324.0633；306.0569；280.0793；262.0647；213.0337
22	10.61	545.2165 [M+Na]+	562.2203	0.835	$C_{32}H_{34}O_9$	Antibiotic ES 242-5	Lignan	co-culture	310.0989；292.0837；274.0726

Table S4. **^{1}H (600 MHz) and ^{13}C (125 MHz) NMR data in $CDOD_3$**

Position	Compound **N5**	
	δ_C, mult. (*J* in Hz)	δ_H, mult. (*J* in Hz)
1	173.3, C	
2	38.5, CH_2	2.45, dd, (16.2,2.6)<br>2.22, dd (16.2, 10.9)
3	72.9, CH	3.91 dt (10.2,4.2)
4	36.8, CH	1.80, m
5	44.8, CH_2	1.19, m<br>0.95, m
6	28.5, CH	1.79, m
7	49.6, CH_2	1.03, m<br>0.98, m
8	27.6, CH	1.64, m
9	39.1, CH_2	1.29, m<br>0.71, m
10	35.9, CH	1.82, m
11	73.0, CH	4.19, d (10.2)
12	119.9, C	
13	145.6, CH	6.91, d (11.40)
14	128.8, CH	6.58, dd (14.7, 11.7)
15	140.7, CH	6.34, ddt (15.1, 10.3, 4.9)
16	37.1, CH_2	2.56, m<br>2.49, m
17	71.6, CH	4.94, dt (10.2,3.5)
18	47.3, CH	2.67, m
19	30.7, CH_2	1.98, m<br>1.40, m
20	26.3, CH_2	1.76, m<br>1.84, m
21	32.9, CH_2	1.97, m<br>1.81, m
22	50.4, CH	2.25, dd, (16.1, 2.9)
23	#	#
24	18.4, CH_3	0.86, d (6.6)
25	19.2, CH_3	0.88, d (6.6)
26	20.9, CH_3	0.84, d (6.6)
27	15.4, CH_3	1.03, d (6.6)
28	117.3, C	

#Not observed

Table S5. Putative functions for genes in the compound N5-related gene cluster

Gene ID	Reference genes	Amino Acids	Closest similar protein (% identity/similarity), Accession *No.*	Proposed function in Borrelidin biosynthesis
ctg1_417	ALV82340.1	237	domain-containing protein from *Streptomyces* sp. WAC02707 (99%) WP_260614318.1	
ctg1_416	ALV82324.1	863	hypothetical protein GCM10010308_03260 from *S. vinaceusdrappus* (99%) GHB95545.1	
ctg1_415	ALV82339.1	212	HD domain-containing protein from *Streptomyces* sp. WAC06128 (99%) WP_127442782.1	
ctg1_414	ALV82316.1	329	GlxA family transcriptional regulator from *S. rochei* (98%) WP_270881709.1	
ctg1_413	ALV82327.1	276	alpha/beta hydrolase from *Streptomyces* sp. AN-3 (99%) WP_282392853.1	
ctg1_412	ALV82332.1	168	carboxymuconolactone decarboxylase family protein from *S. vinaceusdrappus* (99%) WP_261700244.1	
ctg1_411	ALV82329.1	323	hypothetical protein GCM10010385_32990 from *S. geysiriensis* (98%) GGY80192.1	
ctg1_410	ALV82314.1 bor2B	264	alpha/beta fold hydrolase from *S. rochei* (100%) WP_307945156.1	starter unit biosynthesis
ctg1_409	ALV82322.1 bor2C	265	SDR family oxidoreductase from *Streptomyces* sp. AVP053U2 (94%) WP_062189319.1	starter unit biosynthesis
ctg1_408	ALV82326.1 bor2D	250	SDR family oxidoreductase *Streptomyces* sp. SGAir0924 (99%) QCR51210.1	starter unit biosynthesis

ctg1_407	ALV82328.1 bor2E	390	dipeptide epimerase from *Streptomyces* sp. CRB46 (99%) WP_199577149.1	starter unit biosynthesis
ctg1_406	ALV82333.1 bor2F	272	alpha/beta fold hydrolase from *S. rochei* (99%) WP_306693450.1	starter unit biosynthesis
ctg1_405	ALV82334.1 bor2G	539	putative acetolactate synthase from *S. rochei* (99%) ALV82334.1	starter unit biosynthesis
ctg1_404	ALV82343.1 bor2H	675	putative dehydratase from *S. parvulus* (100%) CAE45666.1	starter unit biosynthesis
ctg1_403	ALV82318.1 bor2A1	838	borrelidin type I polyketide synthase from *S. rochei* (100%) WP_307945151.1	PKS module 1(AT-ACP)
ctg1_402	ALV82346.1 bor2A2	808	Borrelidin type I polyketide synthase from *S. parvulus* (97%) CAE45668.1	PKS module2 (KS-AT)
ctg1_399	ALV82320.1 bor2A3	805	Borrelidin type I polyketide synthase from S. parvulus (99%) CAE45672.1	PKS module5,6 (KS-AT)
ctg1_401	ALV82341.1 bor2A4	2514	Borrelidin type I polyketide synthase from *S.parvulus* (98%) CAE45669.1	PKS module2, 3 (DH-KR-ACP-KS-AT-KR-ACP)
ctg1_400	ALV82345.1 bor2A5	2130	Borrelidin type I polyketide synthase from *S. rochei* (100%) WP_307945147.1	PKS module4 (KS-AT-DH-ER-KR-ACP)
ctg1_398	ALV82335.1 bor2A6	858	Borrelidin type I polyketide synthase from *S. rochei* (100%) WP_208949388.1	PKS module5,6 (KR-ACP)
ctg1_396	ALV82330.1 bor2I	396	putative cytochrome P450 from *S. parvulus* (99%) CAE45673.1	nitrile biosynthesis
ctg1_395	ALV82319.1 bor2J	454	aspartate aminotransferase family protein [*S. griseocarneus*] (86%) WP_190066207.1	nitrile biosynthesis
ctg1_394	ALV82323.1 bor2K	326	putative oxidoreductase from *S. rochei* (100%) ALV82323.1	unknown
ctg1_393	ALV82337.1	556	hypothetical protein from *S. parvulus* (99%)	starter unit

	bor2L		CAE45676.1	biosynthesis
ctg1_392	ALV82315.1 bor2M	305	TIGR03619 family F420-dependent LLM class oxidoreductase from *S. caniferus* (91%) WP_159478915.1	starter unit biosynthesis
ctg1_391	ALV82347.1 bor2N	248	fumarylacetoacetate hydrolase family protein from *S. caniferus* (98%) WP_159478917.1	starter unit biosynthesis
ctg1_390	ALV82331.1 bor2O	675	threonine--tRNA ligase from *S. griseocarneus* (99%) WP_190066203.1	borrelidin resistance
ctg1_389	ALV82344.1	122	hypothetical protein from *S. parvulus* (100%) CAE45680.1	
ctg1_388	ALV82338.1	127	STAS domain-containing protein from *S. rochei* (100%) WP_086877709.1	
ctg1_387	ALV82342.1	80	hypothetical protein from *S. rochei* (100%)	
ctg1_386	ALV82317.1	70	hypothetical protein from *S. rochei* (100%) WP_307945134.1	
ctg1_385	ALV82325.1	149	hypothetical protein from *S. parvulus* (100%) CAE45684.1	
ctg1_384	ALV82336.1	160	GNAT family N-acetyltransferase from *S. rochei* (100%) WP_307945131.1	
—	ALV82321.1	—	—	—
ctg1_383	—	675	NAD-binding lipoprotein from S. vinaceusdrappus (94%) WP_261700586.1	
ctg1_382	—	165	hemerythrin domain-containing protein from *S. rochei* (99%) WP_270881716.1	

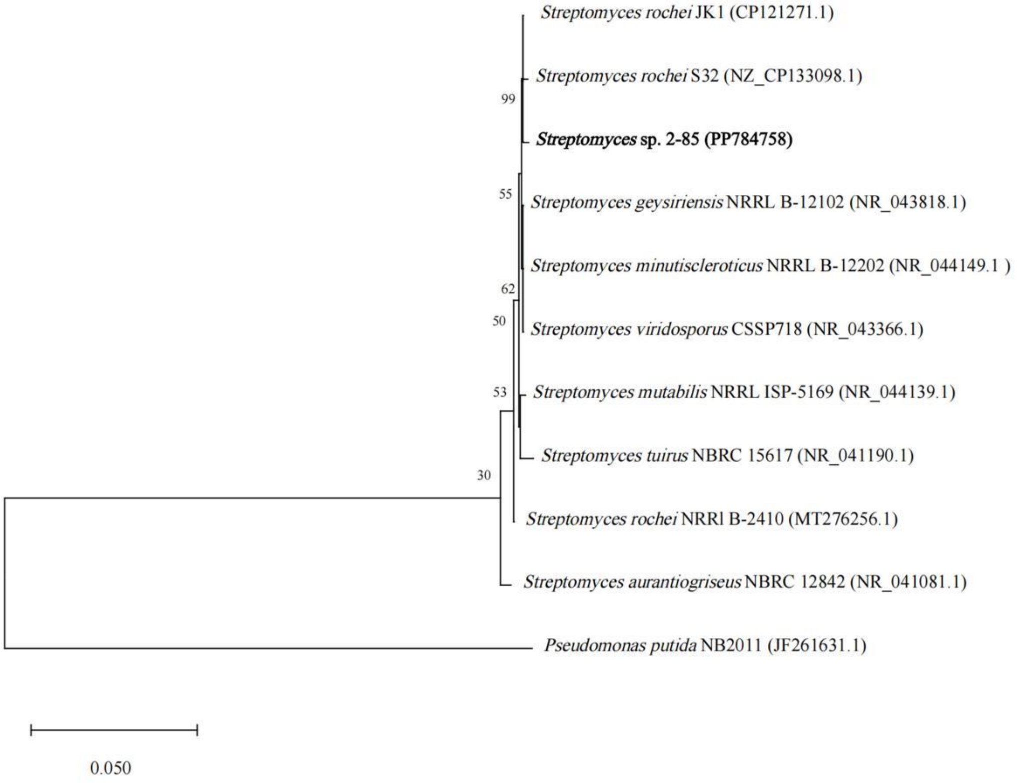

Figure S1. Phylogenetic analysis of *Streptomyces* sp. 2-85 based on the Neighbor-Joining (NJ) method. Bootstrap values based on 1000 replicates are shown at tree nodes. The scale bar at the bottom of the tree indicates K2P genetic distance.

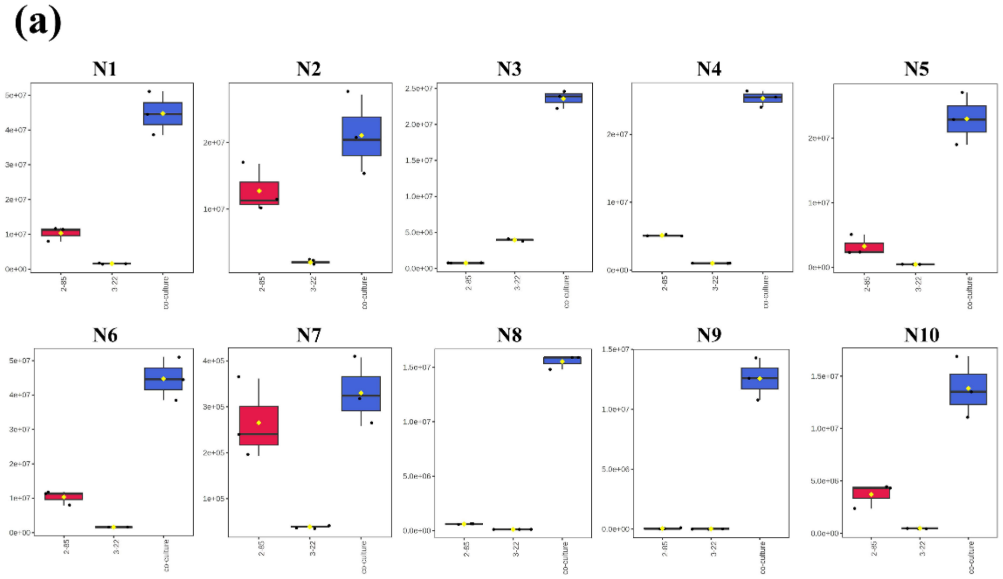

(b)

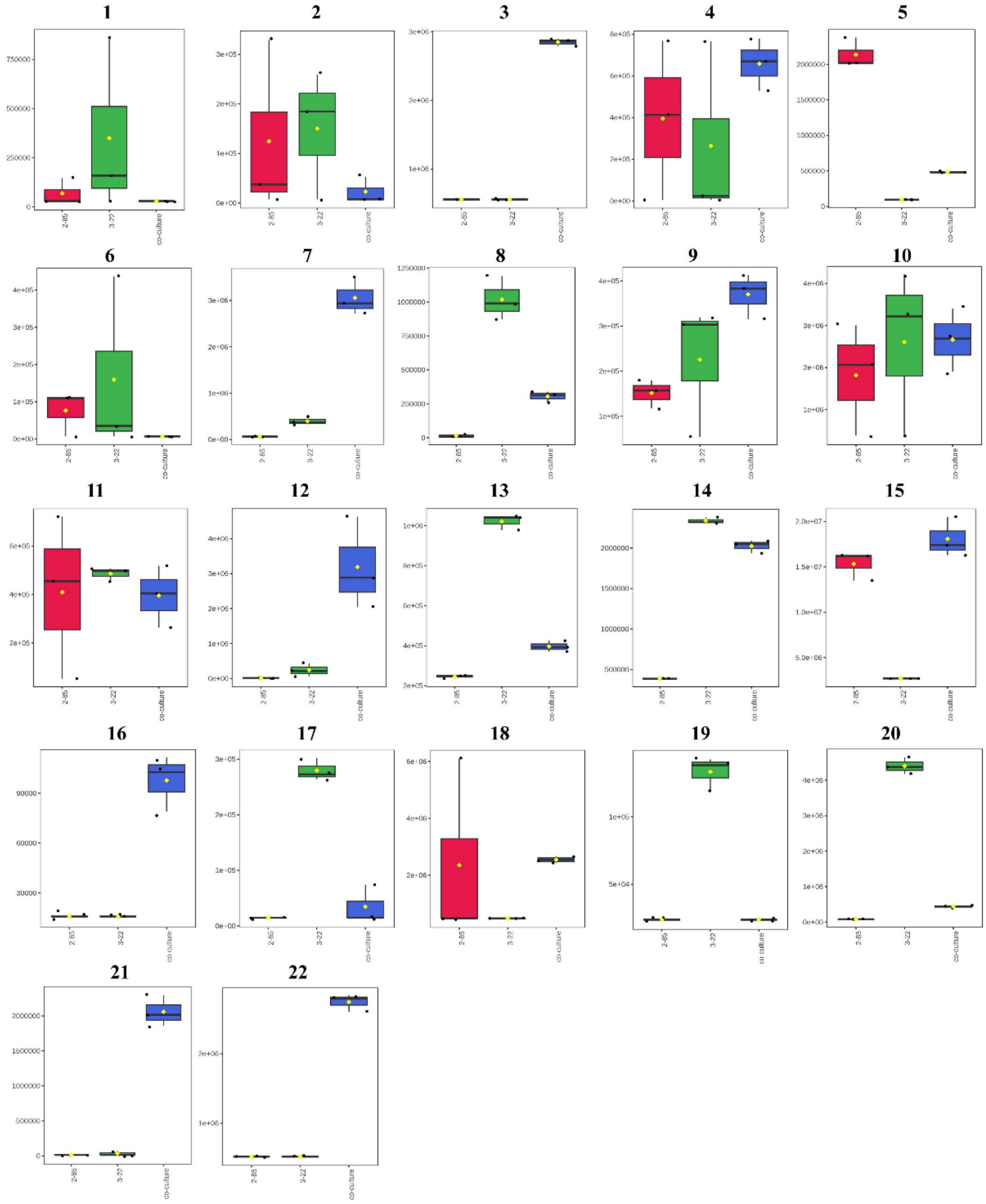

Figure S2. (a, b) The changes in the peak area (AUC) of annotated compounds of strain *Streptomyces* sp. 2-85 and *Cladosporium* sp. 3-22 under co-culture conditions

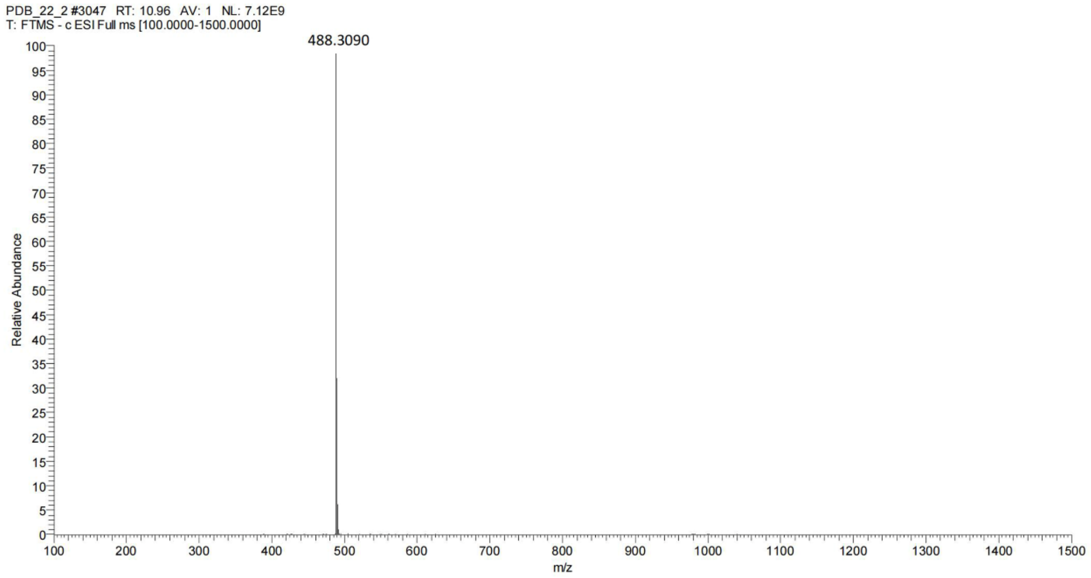

Figure S3. ESI-HRMS mass spectra of compound N5 in negative ion mode

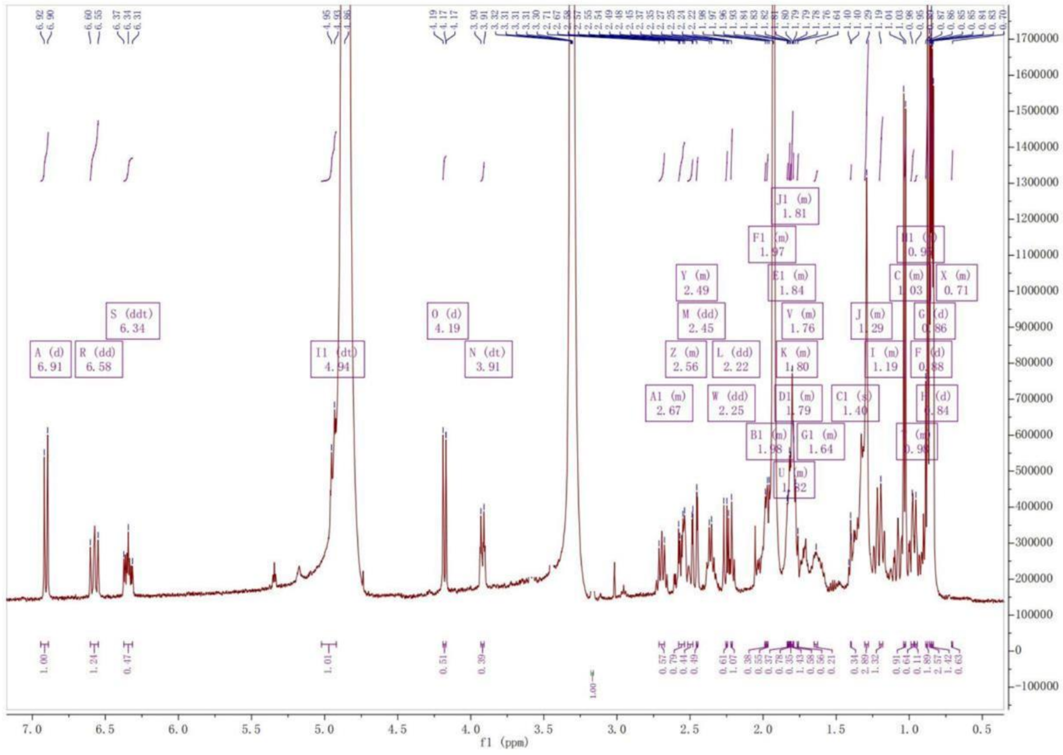

Figure S4. ^{1}H NMR spectrum of compound N5 (600 MHz, $CDOD_3$)

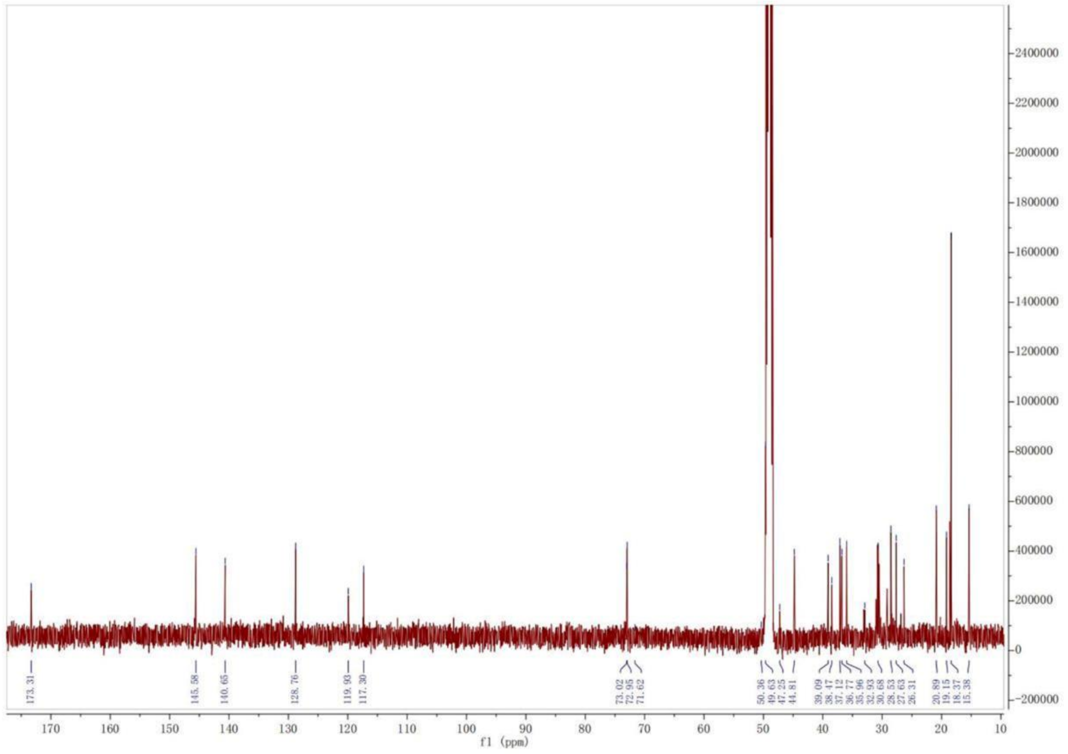

Figure S5. ^{13}C NMR spectrum of compound N5 (600 MHz, $CDOD_3$)

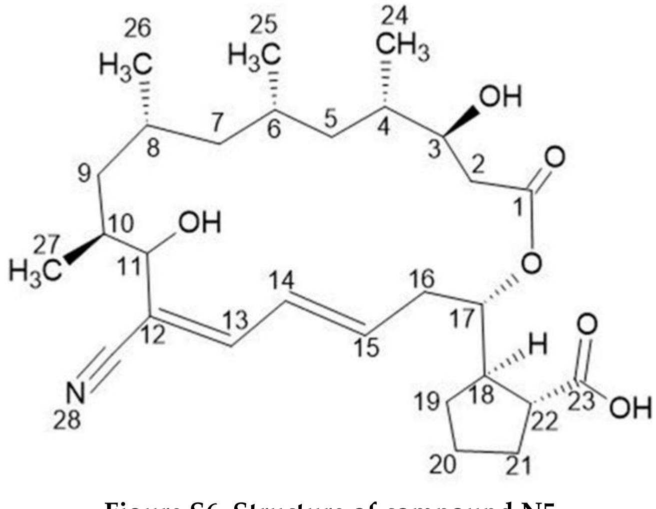

Figure S6. Structure of compound N5
